# Plant‐produced SARS‐CoV‐2 antibody engineered towards enhanced potency and in vivo efficacy

**DOI:** 10.1111/pbi.14458

**Published:** 2024-11-19

**Authors:** Steven W. de Taeye, Loïc Faye, Bertrand Morel, Angela I. Schriek, Jeffrey C. Umotoy, Meng Yuan, Natalia A. Kuzmina, Hannah L. Turner, Xueyong Zhu, Clemens Grünwald‐Gruber, Meliawati Poniman, Judith A. Burger, Tom G. Caniels, Anne‐Catherine Fitchette, Réjean Desgagnés, Virginie Stordeur, Lucie Mirande, Guillaume Beauverger, Godelieve de Bree, Gabriel Ozorowski, Andrew B. Ward, Ian A. Wilson, Alexander Bukreyev, Rogier W. Sanders, Louis‐Philippe Vezina, Tim Beaumont, Marit J. van Gils, Véronique Gomord

**Affiliations:** ^1^ Department of Medical Microbiology and Infection Prevention, Laboratory of Experimental Virology Amsterdam UMC location University of Amsterdam Amsterdam The Netherlands; ^2^ Amsterdam institute for Immunology and Infectious Diseases, Infectious Diseases Amsterdam The Netherlands; ^3^ ANGANY Innovation, 1 voie de l'innovation, Pharmaparc II Val de Reuil France; ^4^ Department of Integrative Structural and Computational Biology The Scripps Research Institute La Jolla California USA; ^5^ Department of Pathology University of Texas Medical Branch Galveston Texas USA; ^6^ Galveston National Laboratory Galveston Texas USA; ^7^ BOKU Core Facility Mass Spectrometry Universität für Bodenkultur Wien Vienna Austria; ^8^ ANGANY Inc Québec Quebec Canada; ^9^ The Skaggs Institute for Chemical Biology, The Scripps Research Institute La Jolla California USA; ^10^ Department of Microbiology and Immunology University of Texas Medical Branch Galveston Texas USA; ^11^ Department of Microbiology and Immunology Weill Cornell Medicine New York New York USA

**Keywords:** antibody, SARS‐CoV‐2, structure, engineering, glycosylation, effector function

## Abstract

Prevention of severe COVID‐19 disease by SARS‐CoV‐2 in high‐risk patients, such as immuno‐compromised individuals, can be achieved by administration of antibody prophylaxis, but producing antibodies can be costly. Plant expression platforms allow substantial lower production costs compared to traditional bio‐manufacturing platforms depending on mammalian cells in bioreactors. In this study, we describe the expression, production and purification of the originally human COVA2‐15 antibody in plants. Our plant‐produced mAbs demonstrated comparable neutralizing activity with COVA2‐15 produced in mammalian cells. Furthermore, they exhibited similar capacity to prevent SARS‐CoV‐2 infection in a hamster model. To further enhance these biosimilars, we performed three glyco‐ and protein engineering techniques. First, to increase antibody half‐life, we introduced YTE‐mutation in the Fc tail; second, optimization of *N*‐linked glycosylation by the addition of a C‐terminal ER‐retention motif (HDEL), and finally; production of mAb in plant production lines lacking β‐1,2‐xylosyltransferase and α‐1,3‐fucosyltransferase activities (FX‐KO). These engineered biosimilars exhibited optimized glycosylation, enhanced phagocytosis and NK cell activation capacity compared to conventional plant‐produced S15 and M15 biosimilars, in some cases outperforming mammalian cell produced COVA2‐15. These engineered antibodies hold great potential for enhancing *in vivo* efficacy of mAb treatment against COVID‐19 and provide a platform for the development of antibodies against other emerging viruses in a cost‐effective manner.

## Introduction

From the start of COVID‐19 pandemic, substantial efforts have been made to develop therapeutics against SARS‐CoV‐2. Most of these efforts have focused on identifying vaccines based on the SARS‐CoV‐2 spike protein that would induce host immune responses and elicit protective antibody responses in healthy individuals.

However, alternative approaches have centered around the administration of monoclonal antibodies (mAbs) that neutralize SARS‐CoV‐2. This passive immunotherapy offers a relatively short‐term but immediate protection against COVID‐19 preventing severe disease (Farhangnia *et al*., 2022; Yu *et al*., 2023). Research groups have intensely focused on pre‐clinical testing of these broadly reactive and potent neutralizing mAbs since the beginning of the pandemic. Many neutralizing antibodies have been identified, most of them targeting the receptor‐binding domain (RBD) of the SARS‐CoV‐2 S1 spike subunit (Brouwer *et al*., 2020; Chen *et al*., 2020; Ju *et al*., 2020) and at least a dozen anti‐SARS‐CoV‐2 mAbs entered clinical trials (Yu *et al*., 2023). But due to the emergence of new circulating viral variants, many, if not all, of the mAbs discovered at the beginning of the pandemic are unable to neutralize current Omicron variants (Cowan *et al*., 2023; Wang *et al*., 2023; Yuan *et al*., 2021). Encouragingly, recent discovery of novel pan‐neutralizing SARS‐CoV‐2 antibodies might enable the utilization of these mAbs for prevention or treatment in patients at risk of severe disease progression (de Campos‐Mata *et al*., 2024; Wang *et al*., 2024).

Irrespective of the clinical efficacy of current approved mAb treatments, the production costs and market price of these therapies are generally too high for broad, world‐wide distribution and administration. One potential solution to decrease pricing is to find alternative production pathways for the expensive mammalian cell culture systems. More than 50 different antibodies have been expressed in various plant expression systems since the first report of antibody production in Tobacco plants (Hiatt *et al*., 1989). Leveraging plant expression platforms can substantially reduce production costs (Nandi *et al*., 2016). For instance, the production of an immune checkpoint inhibitor in plants was calculated to reduce costs by 15–75 times compared to mammalian cell production (Ridgley *et al*., 2023). Furthermore, it is well documented that antibody production in plants using transient expression is both rapid and yields high quantities (Faye *et al*., 2024; Tschofen *et al*., 2016). Nevertheless, a significant challenge in achieving commercial‐scale production of plant‐made pharmaceuticals, including plant‐made antibodies, lies in the complexity and cost associated with purifying them from plant extracts (Tschofen *et al*., 2016). For example, a critical concern during downstream processing of plant‐made biopharmaceuticals, as opposed to other production systems, involves efficiently clarifying the plant extract to swiftly eliminate fibrous particles, plant pigments, and phenolic compounds. Clarification of plant extract combines centrifugation and filtration by depth and pre‐coated filters. We have previously demonstrated that antibodies transiently expressed in *N. benthamiana* can be easily purified from plants using a one‐step magnetic purification protocol (Faye *et al*., 2024).

In this study, we compared COVA2‐15 plant‐made biosimilars with the original human antibody produced in mammalian cells and found that the plant‐produced biosimilars exhibited similar neutralization capacity *in vitro* and equal prevention of disease progression *in vivo* in comparison to mammalian cell produced COVA2‐15 (Brouwer *et al*., 2020). Furthermore, we used glyco‐ and protein engineering strategies to increase half‐life and Fc effector function of plant‐made COVA2‐15 antibodies and characterized two glyco‐engineering strategies that yielded antibodies with enhanced Fc effector functions. The relatively low production costs in combination with the glyco‐engineering approaches to enhance Fc function make plant‐produced monoclonal antibodies a highly advantageous platform for mAb therapy against SARS‐CoV‐2 and other infectious diseases.

## Results

### Interaction of COVA2‐15 with SARS‐CoV‐2 spike

**Figure 1:**
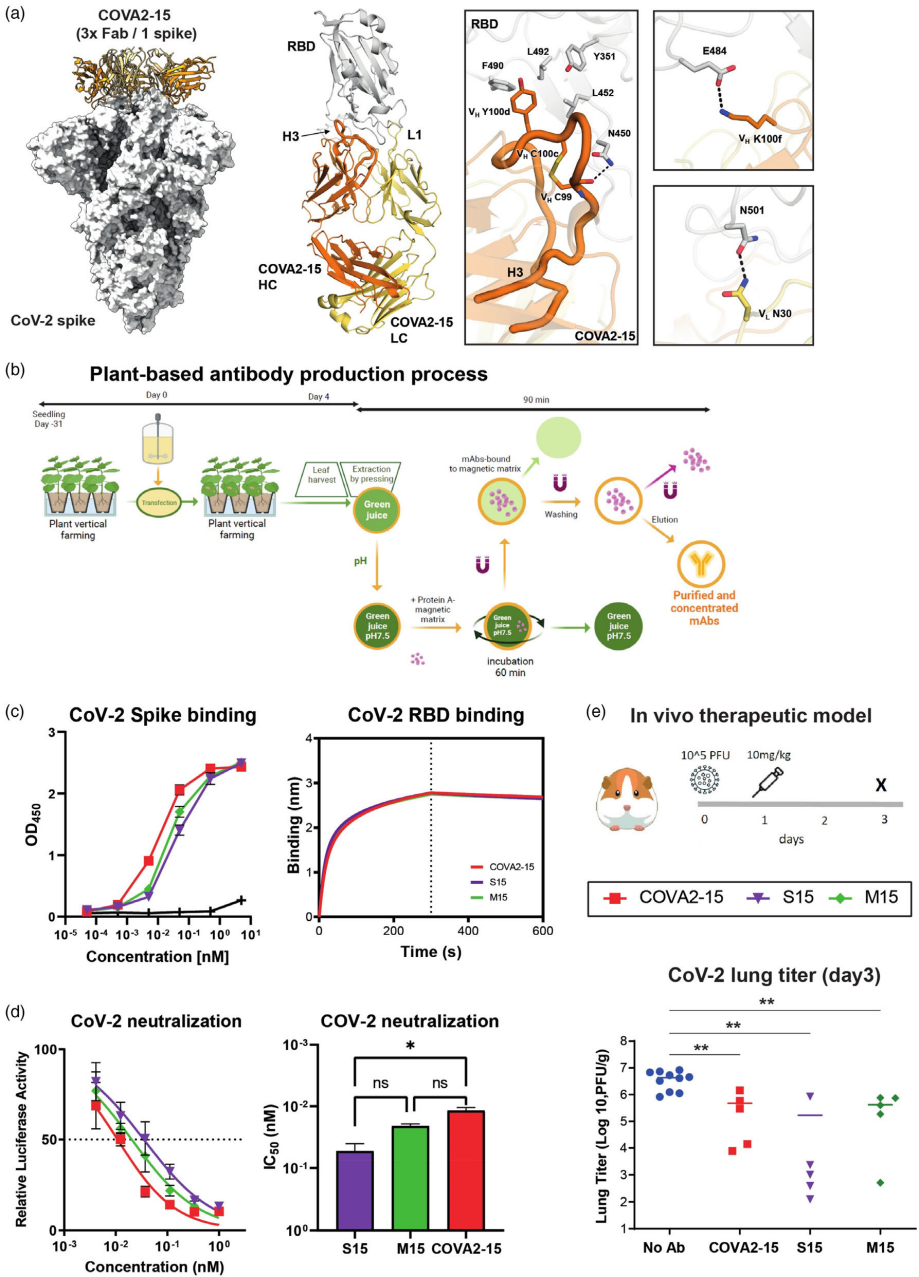
Therapeutic activity of plant‐produced SARS‐CoV‐2 antibody COVA2‐15. (a) Structural information on interaction between human COVA2‐15 antibody and SARS‐CoV‐2 spike protein. The left panel shows a 3.9 Å cryo‐EM structure of three COVA2‐15 Fab domains in complex with the Wuhan SARS‐CoV‐2 6P‐mut7 spike (PDB: 9aru). SARS‐CoV‐2 spike is depicted in a surface representation and COVA2‐15 Fab domains are depicted as orange (HC) and yellow (LC) ribbon cartoon. Middle and right panels show the crystal structure (PDB: 9B82) of COVA2‐15 in complex with the RBD of Wuhan spike, where critical contact residues in CDRH3 are displayed in a stick format. Two separate panels illustrate the interaction of COVA2‐15 with E484 and N501, residues subject to escape mutations in emerging variants after Wuhan. (b) Work‐flow of production of antibodies in *N. benthamiana* plants. Four days post transfection, leaves are harvested and the green juice is subjected to a one‐step Protein A magnetic purification strategy to isolate the plant‐produced antibodies. (c) Binding of human and plant‐produced antibodies (S15/M15) to SARS‐CoV‐2 Spike (Wuhan) and RBD (Wuhan) as determined by ELISA or BLI, respectively. One representative antibody titration experiment shows binding of antibodies to SARS‐CoV‐2 in ELISA. Data from one representative BLI experiment are plotted in which antibodies were used at a concentration of 100 nM. (d) SARS‐CoV‐2 neutralization by plant and human produced COVA2‐15 variants using an antibody serial dilution with a start concentration of 1 nM. Right panel shows mean IC_50_ values ±SD of at least three individual measurements. Statistical differences are determined using an ordinary one‐way ANOVA corrected with a Kruskal–Wallis multiple comparison test and are indicated with asterisks, *P < 0.05, **P < 0.01. (e) Schematic representation of the experiment: Syrian hamsters were infected via IN route with 10 000 PFU of SARS‐CoV‐2, treated via IP route with 10 mg/mL of COVA2‐15, M15, S15 or no Ab treatment on Day 1 and euthanized on Day 3; Lungs viral load on day 3 post challenge, *P*‐values are shown for significant difference. Statistical differences are determined using an ordinary one‐way ANOVA with Tukey's multiple comparisons test and are indicated with asterisks, **P < 0.01.

SARS‐CoV‐2 spike‐specific neutralizing antibodies were isolated previously from convalescent COVID‐19 patients (Brouwer *et al*., 2020), identifying COVA2‐15 with very high neutralization potency. To shed light on the neutralizing potency, the crystal structure of COVA2‐15 Fab domain with wild‐type spike (Wuhan) RBD domain was determined at 3.4 Å (Figure 1a; Table S1). We also obtained a 3.9 Å cryo‐EM structure showing the 3:1 stoichiometry of COVA2‐15 in complex with SARS‐CoV‐2 spike (Figure 1; Table S2; Figure S1a). The cryo‐EM structure reveals that COVA2‐15 binds with all three RBD in the down conformation, at least with respect to the 6P‐mut7 stabilized trimer used in the experiment and belongs to class II RBD targeting antibodies (Barnes *et al*., 2020; Chen *et al*., 2023). COVA2‐15 interacts with the RBD domain through a long CDRH3 and makes additional contacts mainly via a long CDR L1 and minor contacts with L2 (Figure 1a). More specifically, C99‐C100c forms a disulphide bond at the tip of CDRH3 and interacts with N450 in the spike, whereas V_H_ Y100d inserts into a hydrophobic pocket lined by Y352, L452, F490 and L492 in the RBD (Figure 1a). Furthermore, the interaction with E484 and N501 in the RBD domain explains the reduction of neutralization of COVA2‐15 and RBS‐B/ class 2 antibodies (Barnes *et al*., 2020; Yuan *et al*., 2021) in general to SARS‐CoV‐2 variants bearing the E484K/A and N501Y mutations (Figure 1a) (Caniels *et al*., 2021; Jones *et al*., 2021; van Gils *et al*., 2022).

### Binding characteristics and *in vivo* efficacy of plant‐produced COVA2‐15

To generate biosimilars in a cost‐effective manner, we previously expressed COVA2‐15 in wild type *N. benthamiana* plants using two different formats; conventional IgG1: M15 and a single‐chain (sc)Fv‐Fc format: S15 (Figure 1b) (Faye *et al*., 2024). The single‐chain format simplifies the production process compared to full IgG, while maintaining the Fc region for easy purification and *in vivo* half‐life. Using a one‐step Protein A magnetic purification strategy, S15 and M15 antibodies were previously isolated from crude plant extracts with high efficiency and purity (production schematic; Figure 1b) (Faye *et al*., 2024). Here, using bio‐layer interferometry (BLI) and ELISA, we analysed binding capacities to spike and RBD of plant‐made M15 and S15 in comparison to HEK‐293F derived COVA2‐15. Both ELISA and BLI analysis using recombinant SARS‐CoV‐2 Spike and RBD protein revealed that the binding kinetics of plant‐produced M15 and S15 closely resembled that of human derived COVA2‐15 (Figure 1c). M15 neutralized SARS‐CoV‐2 with similar potency as HEK‐293F derived COVA2‐15 (Figure 1d). Neutralization capacity of S15 was slightly lower (IC_50_ = 0.053 nM) in comparison to HEK‐293F derived COVA2‐15 (IC_50_ = 0.012 nM) (Figure 1d), suggesting that the orientation and flexibility of the Fab domains with respect to the Fc domain may influence neutralization capacity. Collectively, the binding and neutralization data illustrated that plant‐produced M15 and S15 interact with and neutralize SARS‐CoV‐2 in a manner similar to their COVA2‐15 counterpart expressed in HEK 293F cells.

We then compared the therapeutic efficacy of M15, S15 and COVA2‐15 in an *in vivo* hamster model of SARS‐CoV‐2 infection. A dose of 10 mg/kg antibody was administered intraperitoneal 24 h after intranasal SARS‐CoV‐2 challenge (Figure 1e). By day 3 post infection, the COVA2‐15‐, S15‐ and M15‐treated groups exhibited significantly lower mean lung viral titers compared with the control group (4.2 × 10^6^ PFU/g of lung tissue for control, 0.47 × 10^6^ PFU/g for COVA2‐15, 0.17 × 10^6^ PFU/g for S15 and 0.41 × 10^6^ PFU/g for M15, *P* < 0.01). S15 mAb‐treated animals appeared to exhibit lower viral titer in lungs compared to the other groups; however, the difference between groups receiving mAb treatment was not significant (Figure 1e).

There were no significant differences in terms of weight loss between control animals and those treated with mAbs (Figure S1b). Additionally, no significant variations in serum neutralization capacity were observed among the mAb‐receiving groups. Serum from four out of five animals treated with S15 exhibited greater virus inhibition efficiency compared to the M15‐treated group (Figure S1c). This observation highlights the potential advantages of S15 in terms of pharmacokinetics *in vivo* compared to M15.

### Plant‐produced glyco‐engineered variants show enhanced Fc gamma receptor binding

**Figure 2:**
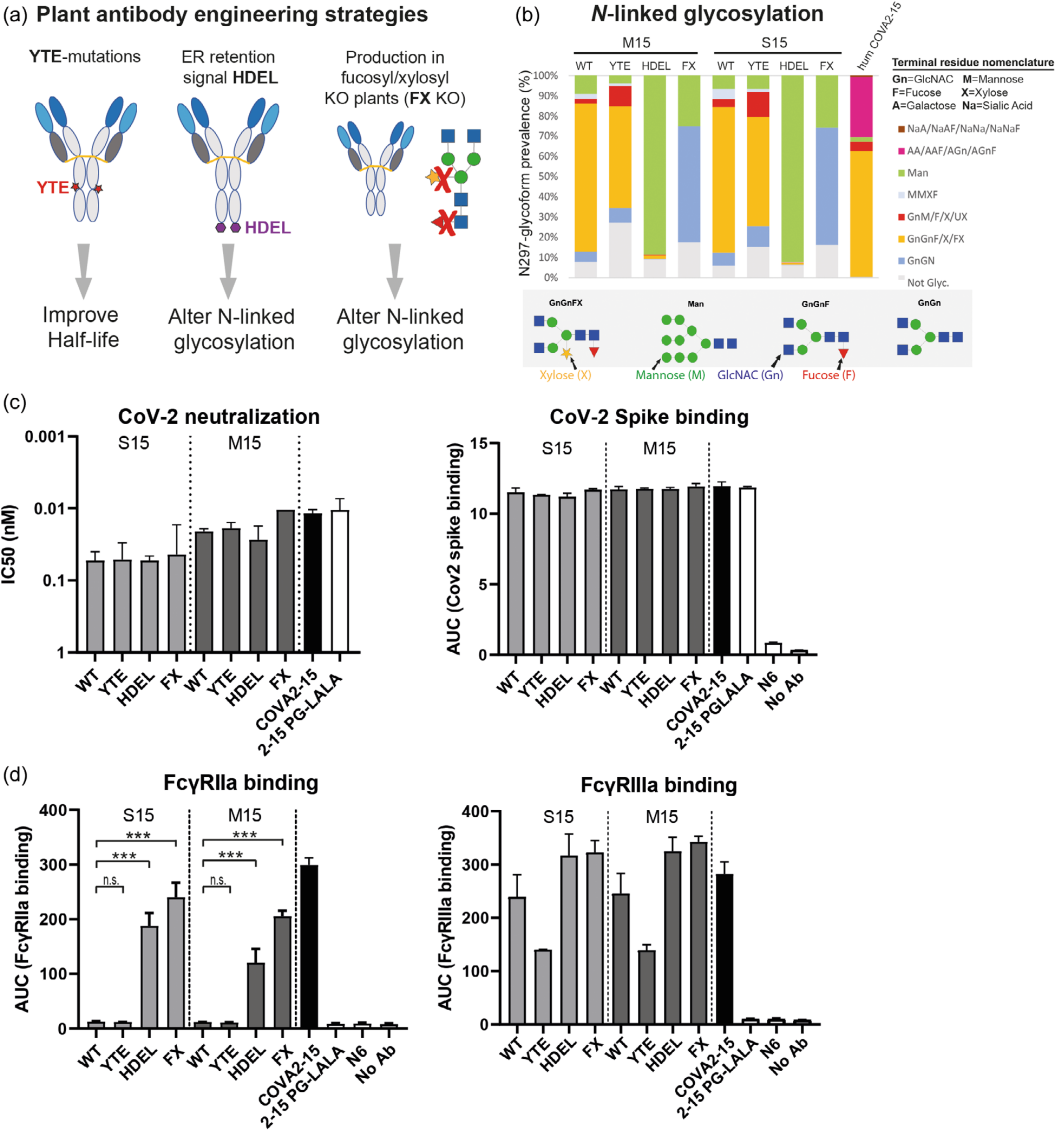
Engineering strategies to produce antibodies in plants with enhanced therapeutic activity. (a) Illustration of three engineering strategies (YTE, HDEL and FX) that were used to further improve plant‐produced COVA2‐15 antibodies. YTE mutations (M252Y/S254T/T256E) are introduced in the Fc domain to enhance Ab half‐life. The C‐terminal HDEL retention signal prevents Golgi trafficking and thereby alters *N*‐linked glycosylation. Production of antibody in fucosyl/xylosyl (FX) transferase knock out plants inhibits fucosylation and xylosylation of antibodies. (b) Depiction of *N*‐linked glycosylation forms of all human and plant‐produced antibody variants as determined by mass spectrometry. A terminal residue based nomenclature is used to specify the *N*‐linked glycoforms (Altmann *et al*., 2024). Coloured bar graphs show the prevalence of each glycoform (or group of glycoforms) for each individual antibody. A schematic illustration of the most prevalent glycoforms is depicted below to enhance visualization. (c) Binding of plant‐produced S15 variants (light grey), plant‐produced M15 variants (dark grey) and mammalian produced COVA2‐15 (black) to SARS‐CoV‐2 Spike (Wuhan) as determined with an IgG ELISA. SARS‐CoV‐2 neutralization by plant and human produced COVA2‐15 antibodies plotted as IC_50_ values. (d) FcγRIIa and FcγRIIIa binding to antigen bound COVA2‐15 variants, as determined by FcγR‐dimer ELISA. AUC values derived from full titration curves (Figure S2) are plotted of at least two individual measurements. Statistical differences are determined using an ordinary one‐way ANOVA corrected with a Dunns multiple comparison test and are indicated with asterisks,   ***P < 0.001.

Different glyco‐ and protein‐Fc engineering strategies were used to further enhance stability, potency and efficacy of plant‐produced M15 and S15. The *N‐*linked glycosylation machinery is different in plants compared to mammalian cells, and thus influences the composition of the *N*‐linked glycan at position 297 in the Fc domain, and thereby modulates antibody stability, immunogenicity, and Fc gamma receptor (FcγR) binding (Damelang *et al*., 2024; de Taeye *et al*., 2019). To minimize plant‐specific glycosylation and optimize Fc function, we generated two families of glyco‐engineered mAbs (Figure 2a). The first strategy makes use of the addition of an endoplasmic reticulum (ER) ‐retention signal (HDEL) to the C‐terminus of the COVA2‐15 heavy chain, which prevents transport to and glycan processing of S15‐HDEL and M15‐HDEL in the Golgi, particularly the addition of core α‐1,3 fucose and β‐1,2‐xylose residues (Gomord *et al*., 2010; Lee *et al*., 2018; Song *et al*., 2021). The second glyco‐engineering strategy involves the production of antibodies in the FX‐KO line of *N. benthamiana*, which is deficient in plant‐specific α‐1,3‐fucosyltransferase and β‐1,2‐xylosyltransferase activities (Göritzer *et al*., 2022; Jansing *et al*., 2019), thereby producing S15‐FX and M15‐FX antibodies deficient for xylose and fucose glycan moieties (Figure 2a). To prolong antibody half‐life and increase their bioavailability, M252Y/S254T/T256E (YTE) mutations were introduced into M15 and S15, subsequently named M15‐YTE and S15‐YTE, respectively (Figure 2a).

As illustrated in Figure S2a, glyco‐ and protein‐Fc engineered variants were purified to electrophoretic homogeneity using protein A magnetic bead purification. The non‐reduced analysis showed that most heavy (H) and light (L) chains were correctly assembled to form LHHL complexes, and only minor cleavage/degradation products (HH and HHL) were detected for plant‐made M15 variants and COVA2‐15 produced in mammalian cells (Figure S2a). Yields expressed as purified mAb per kg of fresh tobacco leaves were similar for all M15 and S15 variants, reaching 24–34 mg/kg. Yield was estimated from twenty different production batches of 0.7–1 kg of leaves. We characterized the *N*‐linked glycosylation profile of the engineered M15 and S15 variants and compared it with conventional plant produced M15 and S15 and mammalian HEK‐293F cell derived COVA2‐15 (Figure 2b). We previously showed that the major glycoforms identified on plant‐produced M15 and S15 are complex type glycans containing α‐1,3 fucose and β‐1,2‐xylose (GnGnXF) (Faye *et al*., 2024). Other glycoforms (Man7‐Man9, GnGn, GnGnF, GnGnX, MMXF and GnMXF, see Figure 2b) were detected as well, although less prevalent (Table S3). In contrast to M15 and S15 produced in wild type *N. benthamiana*, the glyco‐engineered mAbs M15‐FX and S15‐FX are completely devoid of plant‐specific core α‐1,3‐fucose and β‐1,2‐xylose residues and the predominant glycoforms are complex GnGn (52%–54%) and M5‐M9 high mannose type N‐glycans (22%–24%) (Figure 2b). In comparison to WT plant‐produced antibodies, the FX variants show slightly more unglycosylated sites (~15%).

The ER retention signal also had a major influence on *N*‐linked glycosylation, as M15‐HDEL and S15‐HDEL variants showed 88%–92% of high‐mannose‐type N‐glycans and less than 2% of glycans containing α‐1,3‐fucose and β‐1,2‐xylose residues, confirming that HDEL‐mediated ER retention was successful and glycan processing in the Golgi was inhibited. Surprisingly, the YTE mutations also influenced the *N*‐linked glycosylation profile for both M15 and S15, since we observed a larger percentage (>10%) of antibodies for which a glycan at Asn297 was absent. Thus, compared with *N*‐linked glycosylation of human COVA2‐15, where the major glycoform is α‐1,6 fucosylated GnGnF (62%), lack of, or a strongly reduced percentage of fucosylation was observed for M15‐FX, S15‐FX, M15‐HDEL and S15‐HDEL.

The engineered antibodies bound with similar affinity to SARS‐CoV‐2 spike in ELISA and neutralized SARS‐CoV‐2 infection with similar IC_50_ when compared with WT S15 and M15, indicating that the engineering strategies and resulting glycosylation differences did not influence Fab‐binding characteristics and potency (Figure 2c). Next, we determined FcγR binding by the various engineered plant‐produced antibodies in comparison to COVA2‐15. The WT plant produced S15 and M15 did not bind to FcγRIIa and exhibited reduced binding to FcγRIIIa compared to human COVA2‐15, indicating that the plant‐specific *N*‐linked glycosylation pattern is strongly impacting FcγR binding. Glycoengineered plant‐produced HDEL and FX antibodies showed enhanced binding to FcγRIIa and FcγRIIIa (Figure 2d) compared to the WT S15 and M15 antibodies. Due to the absence of a fucose residue in their *N*‐linked glycan, binding of HDEL and FX antibodies to FcγRIIIa was even stronger when compared with COVA2‐15 produced in mammalian cells (Figure 2d). The YTE mutant antibodies showed slightly decreased binding to FcγRs when compared with the WT plant‐produced antibodies, suggesting that YTE mutations allosterically impact FcγR interaction. To summarize, we used two engineering strategies (HDEL and FX) to minimize plant‐specific *N*‐linked glycosylation of COVA2‐15 biosimilars and thereby achieved interaction with human FcγRIIa and FcγRIIIa in line with properties of COVA2‐15 produced in mammalian cells.

### Plant‐produced COVA2‐15 variants improve Fc effector function

**Figure 3:**
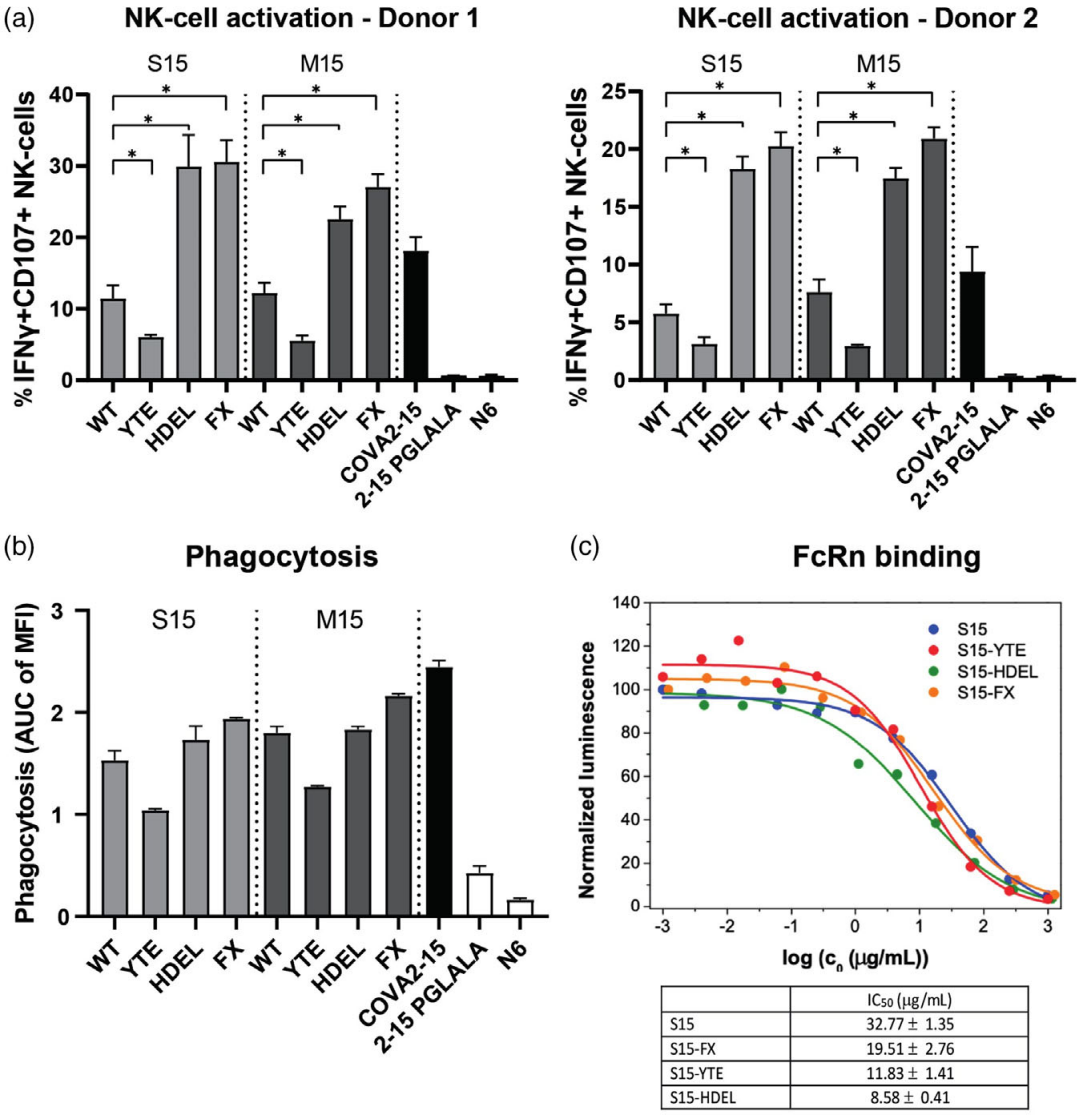
Fc effector function of glyco‐engineered plant produced COVA2‐15 antibodies. (a) NK cell activation by plant‐produced S15 variants (light grey), plant‐produced M15 variants (dark grey) and mammalian produced COVA2‐15 (black) is plotted as the percentage of CD107+ IFNγ+ double‐positive NK‐cells after incubation with antigen‐bound antibodies in a plate based NK cell activation assay. NK cell activation was determined with MACS negatively selected NK cells from two healthy blood donors. Statistical differences are determined using a Mann–Whitney *t*‐test and are indicated with asterisks, *P < 0.05. (b) Antibody dependent cellular phagocytosis (ADCP) determined by the internalization of SARS‐CoV‐2 spike coated fluorescent beads in the presence of plant‐produced S15 variants (light grey), plant‐produced M15 variants (dark grey) and mammalian produced COVA2‐15 (black). AUC values derived from full titration curves (Figure S3c) are plotted from two individual measurements. (c) Binding of S15 variants to the neonatal Fc receptor (FcRn) as determined with an FcRn‐SmBiT luminescence assay. Normalized luminescence was calculated by assigning 100% to maximum signal in the absence of an analyte and then calculating percentage of maximum signal in the presence of an analyte. Mean IC_50_ values ±standard deviation of two individual measurements is depicted.

To see whether the differences in FcγR binding correlated to altered Fc effector function of the plant‐produced antibodies, we determined NK cell activation (FcγRIIIa mediated) and phagocytosis (predominantly FcγRIIa mediated) in comparison to the human COVA2‐15 antibody (Figure 3a). HDEL and FX engineered variants showed 3‐fold enhanced NK cell activation compared to the WT plant antibody, as measured by the expression of CD107 and IFNγ. The level of activation even outperformed the human monoclonal COVA2‐15 (Figure 3a), probably due to the lower fucosylation levels of the engineered Abs. The magnitude of activation correlated well with FcγRIIIa binding strength in ELISA (Figure S3b). The YTE mutant antibodies, in line with reduced FcγRIIIa binding, also showed reduced NK cell activation capacity (Figure 3a). In parallel, we analysed CD16 shedding in the NK cell activation assay and found that NK cell activation is correlated with CD16 shedding (Figure S3). The capacity to induce phagocytosis was determined by opsonizing SARS‐CoV‐2 coated fluorescent beads and quantification of bead internalization by THP‐1 cells using flow cytometry. Here, in line with reduced FcγRIIa binding, we observed a reduced phagocytosis capacity with YTE mutant antibodies. HDEL antibodies, and especially FX antibodies, showed slightly improved phagocytosis compared to WT plant antibodies (Figure 3b). However, mammalian cell produced COVA2‐15 still outperformed the plant‐produced engineered variants in terms of phagocytosis activity, suggesting that the glycosylation profile of the engineered variants is not yet optimal for ADCP or lacks homogeneity.

The S15‐YTE mutant showed increased binding to the neonatal Fc receptor FcRn compared to S15 WT, indicating that this mutant might have a longer half‐life *in vivo* (Figure 3c). Unexpectedly, FcRn binding was also enhanced for the S15‐HDEL antibody, suggesting that the high mannose glycosylation profile might influence FcRn binding and half‐life (Figure 3c).

Thus, glyco‐engineering strategies improve Fc effector function of a SARS‐CoV‐2 antibody and therefore plant‐produced anti‐SARS‐CoV‐2 antibodies have the potential to be developed as a cost‐effective (preventive) therapy for patients at risk.

## Discussion

There is a broad interest in recombinant antibody production for therapeutic application in auto‐immune diseases, cancer and infectious diseases due to the specificity, safety and pharmacokinetics of mAbs. Pharmaceutical companies are continuously seeking for alternatives to bioreactor‐based GMP production and cost reduction for downstream processing of mAb production. Plant‐based antibody production has demonstrated to be a powerful and cost‐effective alternative to produce recombinant antibodies with high yield and purity (Nandi *et al*., 2016; Ridgley *et al*., 2023). Recent achievements have contributed to increasing yields and simplification of upstream and primary recovery processes (Diamos *et al*., 2020) reducing overall costs and improving turn‐over time and quality of the final product. Recently, in the context of the COVID 19 pandemic, several plant‐made mAbs were shown to neutralize SARS‐CoV‐2 *in vitro* to a similar extent as their human orthologues (Frigerio *et al*., 2022; Rattanapisit *et al*., 2020). Consistent with these studies, we found that plant‐made mAbs, M15, and scFv‐Fc, S15, show similar neutralizing activities *in vitro* as their homologue COVA2‐15 previously produced in HEK293T mammalian cells (Brouwer *et al*., 2020) and significantly reduce SARS‐CoV‐2 infection in a hamster model of COVID‐19.

In the current study, the focus was not on increasing total yield (Diamos *et al*., 2020; Jugler *et al*., 2023; Zischewski *et al*., 2016) but to demonstrate that the quality of plant produced mAbs match mammalian cell‐culture produced antibodies. For this, an expression cassette originally developed for the production of eBioparticles™ (Castenmiller *et al*., 2023) has been used which gives 10‐20X less yield (approximately 50 mg per kg of leaf material) compared to optimized expression cassettes (Diamos *et al*., 2020; Nandi *et al*., 2016; Ridgley *et al*., 2023). Notwithstanding, the use of the optimized cassettes would increase the production of COVA2‐15.

High levels of CoV‐2 specific afucosylated antibodies in COVID‐19 hospitalized patients were found to contribute to hyperinflammation and disease severity, therefore antibody therapy is considered disadvantageous in this stage of disease (Hoepel *et al*., 2020; Larsen *et al*., 2021). Therefore, prevention of life‐threatening SARS CoV‐2 disease by antibody therapy must be in a prophylactic administrative setting (Focosi *et al*., 2022). Early‐stage Ab treatment studies demonstrated the contribution of Fc effector functions to the *in vivo* therapeutic efficacy of SARS‐CoV‐2 mAbs (Grandits *et al*., 2023; Moore *et al*., 2021; Yang *et al*., 2023). Additionally, Fc engineered anti‐CoV‐2 mAbs were found to have similar efficacy at a dose of 1 mg/kg compared to conventional mAb treatment at 5 mg/kg (Yamin *et al*., 2021). This illustrates that a functional Fc domain is advantageous for the efficacy and cost‐effectiveness of mAb therapy. Given that plant‐produced antibodies harbour plant‐specific glycosylation, Fc effector function is often impaired. We investigated the Fc effector function of plant‐produced COVA2‐15 and used glyco‐engineering strategies to modify plant‐specific glycosylation forms and thereby improve therapeutic efficacy *in vivo*. Making use of an ER retention signal strategy or *N. benthamiana* plants deficient in xylosyltransferase and fucosyltransferase activity, we generated plant‐produced COVA2‐15 Abs with enhanced effector functions (HDEL and FX variants) compared to conventional plant‐produced COVA2‐15. To maximize the therapeutic potential of future anti‐SARS‐CoV‐2 mAbs that are both cost‐effective and have strong Fc functionality, glyco‐engineered plant‐produced antibodies offer many advantages. The production of antibodies in xylosyl‐ and fucosyl‐transferase knock‐out plants has been explored for multiple infectious diseases such as HIV, Ebola, Zika and SARS‐CoV‐2 (Anand *et al*., 2021; Frigerio *et al*., 2022; Grandits *et al*., 2023; Moore *et al*., 2021; Yang *et al*., 2023). As previously shown with a cocktail of mAbs for post‐exposure treatment of Ebola virus using passive immunotherapy (Prevail II Writing Group, n.d.; Zhang *et al*., 2014) the present study further illustrates the robustness and versatility of the plant expression system for fast production of therapeutic antibodies with potent Fc effector functions in case of new emerging infectious diseases.

COVA2‐15 was one of the most potent neutralizing antibodies against the original Wuhan CoV‐2 variant (Brouwer *et al*., 2020). Accumulation of mutations in the spike protein has resulted in a loss of neutralizing capacity against new emerging variants of concern, that is evident from the molecular interactions determined by cryo‐EM and crystallography (Jones *et al*., 2021; Yuan *et al*., 2021). When the neutralizing activity of mAbs decrease due to emergence of new viral variants, Fc effector functions are crucial to maintain some level of cross‐clade protection against SARS‐CoV‐2 (Mackin *et al*., 2023; Paciello *et al*., 2024; Zhang *et al*., 2023), again highlighting the contribution of the Fc domain to in vivo efficacy of anti‐SARS‐CoV‐2 antibodies.

The efficacy of mAb therapy in SARS‐CoV‐2‐infected individuals is dependent on the antibody half‐life and tissue distribution. Antibody half‐life extension strategies can increase the longevity of the antibodies and, hence, the antibody levels in the body and reduce the number of antibody administrations per patient. Our plant‐produced COVA2‐15 harbouring the YTE mutation‐set increased binding to the neonatal Fc receptor and is known to increase antibody half‐life. However, the allosteric effect of the YTE mutations on Fc gamma receptor binding and Fc effector functions that we observed in this study indicates that other half‐life extension strategies are probably more effective for production of SARS‐CoV‐2‐specific antibodies (Foss *et al*., 2024). Administration of antibodies through a nasal spray offers an alternative way to increase local antibody concentration and efficacy in the lung. Intranasal administration of plant‐produced COVA2‐15 IgA was recently found to protect from CoV‐2 challenge in a hACE mice model (Göritzer *et al*., 2024).

When mAbs contain an *N*‐linked glycan in the Fab domain (5%–20% of antibodies), plant‐specific *N*‐linked glycosylation could influence antigen binding and antibody stability. Since sialylation of the *N*‐linked Fab glycans has been described to contribute to stability and/or antigen binding (van de Bovenkamp *et al*., 2018a, 2018b), plant‐produced biosimilars of such mAbs should be characterized carefully as plants lack the sialylation machinery (Gomord *et al*., 2010). However, the majority of mAbs, including COVA2‐15, do not bind their antigen in a glycan‐dependent manner and therefore binding and neutralization capacity of plant‐produced antibodies is likely to be unaltered (as shown here for S15 and M15).

In conclusion, the present study illustrates that *in vivo* efficacy of plant produced antibody can be rapidly optimized using transient expression, magnetic purification, and protein‐ and glycoengineering strategies. Furthermore, it provides a way forward for cost‐effective mAb production for prophylactic and therapeutic antibody treatment strategies against current infectious diseases or future pandemics.

## Materials & methods

### Production of SARS‐CoV‐2 RBD and COVA2‐15 Fab

The expression plasmids for COVA2‐15 Fabs were transiently co‐transfected into ExpiCHO cells at a ratio of 2:1 (HC:LC) using ExpiFectamine™ CHO Reagent (Thermo Fisher Scientific) according to the manufacturer's instructions. The supernatant was collected at 10 days post‐transfection. The Fab was purified with a CaptureSelect™ CH1‐XL Affinity Matrix (Thermo Fisher Scientific) followed by size exclusion chromatography. Expression and purification of the SARS‐CoV‐2 spike receptor‐binding domain (RBD) for crystallization were as described previously (Yuan *et al*., 2020). Briefly, the RBD (residues 333–529) of the SARS‐CoV‐2 spike (S) protein (GenBank: QHD43416.1) was cloned into a customized pFastBac vector (Ekiert *et al*., 2011), and fused with an N‐terminal gp67 signal peptide and C‐terminal His_6_ tag (Yuan *et al*., 2020). A recombinant bacmid DNA was generated using the Bac‐to‐Bac system (Life Technologies). Baculovirus was generated by transfecting purified bacmid DNA into Sf9 cells using FuGENE HD (Promega), and subsequently used to infect suspension cultures of High Five cells (Life Technologies) at an MOI of 5–10. Infected High Five cells were incubated at 28 °C with shaking at 110 r.p.m. for 72 h for protein expression. The supernatant was then concentrated using a 10 kDa MW cutoff Centramate cassette (Pall Corporation). The RBD protein was purified by Ni‐NTA, followed by size exclusion chromatography, and buffer exchanged into 20 mM Tris–HCl pH 7.4 and 150 mM NaCl.

### Crystallization and structural determination

The COVA2‐15/RBD complex was formed by mixing each of the protein components at an equimolar ratio and incubating overnight at 4 °C. The protein complex was adjusted to 15 mg/mL and screened for crystallization using the 384 conditions of the JCSG Core Suite (Qiagen) on our robotic CrystalMation system (Rigaku) at Scripps Research. Crystallization trials were set‐up by the vapour diffusion method in sitting drops containing 0.1 μL of protein and 0.1 μL of reservoir solution. Optimized crystals were then grown in drops containing 0.095 M sodium citrate, pH 5.6, 19% (v/v) 2‐propanol, 5% (v/v) glycerol, and 19% (w/v) polyethylene glycol 4000 at 20 °C. Crystals appeared on day 3, were harvested on day 15 without additional cryoprotectant and then flash cooled and stored in liquid nitrogen until data collection. Diffraction data were collected at cryogenic temperature (100 K) at beamline 23‐ID‐D of the Argonne Photon Source (APS). Diffraction data were processed with HKL2000 (Otwinowski and Minor, 1997). Structures were solved by molecular replacement using PHASER (McCoy *et al*., 2007). Models for molecular replacement of were derived from PBD 7JMO. Iterative model building and refinement were carried out in COOT (Emsley *et al*., 2010) and PHENIX (Adams *et al*., 2010), respectively. Epitope and paratope residues, as well as their interactions, were identified by accessing PISA at the European Bioinformatics Institute (http://www.ebi.ac.uk/pdbe/prot_int/pistart.html) (Krissinel and Henrick, 2007).

### Cryo‐electron microscopy (cryo‐EM)

50 μg of SARS‐CoV‐2 6P‐mut7 spike protein was incubated with 120 μg COVA2‐15 Fab for 3 h at room temperature (RT). The complex was then purified using a Superdex 200 Increase (Cytiva) gel filtration column, and the corresponding trimer:Fab complex peak was concentrated to 4.3 mg/mL. Immediately before freezing, DDM (N‐dodecyl‐β‐D‐maltopyranoside; Anatrace) was added to a final concentration of 0.06 mM to assist with particle tumbling. A 3 μL drop of the mixture was applied to a plasma‐cleaned Quantifoil 1.2/1.3–400 holey carbon grid (Electron Microscopy Sciences), and vitrified in liquid ethane using a Thermo Fisher Scientific Vitrobot Mark IV (100% humidity, 4 °C, 3–5 s blot times).

Data were collected on a Thermo Fisher Scientific Arctica (200 keV) and a Gatan K2 Summit (4 K × 4 K) camera with Leginon software (Cheng *et al*., 2021). The nominal magnification was 36 000× resulting in a pixel size of 1.15 Å. A total of 2770 micrographs were collected in a defocus range of −0.7 to −2.0 μm and an average dose of 50 e^−^/Å^2^ per micrograph. Movies were aligned and dose‐weighted with MotionCor2 (Zheng *et al*., 2017) and imported into cryoSPARC (Punjani *et al*., 2017). CTF corrections were performed using Gctf, particles were picked by the use of a combination of Blob Picker and Template Picker, and subjected to rounds of 2D classification, Ab Initio 3D modelling, Heterogenous Refinement, and Non‐Uniform Refinement (with global CTF corrections). C3 symmetry was applied to the final refinement of 25 728 particle images, resulting in an estimated ~3.9 Å global FSC (0.143) resolution (Table S1).

Model building was initiated by fitting the COVA2‐15 Fab/RBD co‐crystal coordinates into the cryo‐EM map and using PDB 6vsb as the template for the remainder of the Spike protein in UCSF Chimera (Pettersen *et al*., 2004). The complete model was then refined iteratively with Coot (Casañal *et al*., 2020), Phenix real space refine (Afonine *et al*., 2018), and Rosetta Relax (Conway *et al*., 2014). Final validation was performed with the MolProbity (Williams *et al*., 2018) and EMRinger (Barad *et al*., 2015) implementations in Phenix, and statistics are summarized in Table S1. The map and model have been deposited to the Electron Microscopy Data Bank and Protein Data Bank with accession codes summarized in Table S1.

### Plant antibody production

#### Molecular design, cDNA assemblies and preparation of plasmids

For the expression of recombinant M15 mAb and its scFv‐Fc homologue (S15), the cDNA encoding these antibodies (accession number: QKQ15273 and QKQ15189.1), was fused at the C‐terminus to the tobacco chitinase signal sequence (accession number: QEQ12695) as described before (Faye *et al*., 2024). HDEL variants for both M15 and S15 present C‐terminal ER retention signal. YTE variants were also designed, and they correspond to the combination of three mutations (M252Y/S254T/T256E) (Dall'Acqua *et al*., 2006). The sequences of all cDNAs described above were codon optimized for expression in Nicotiana benthamiana (Geyer *et al*., 2010) at the 5′ and 3′ ends of each of the cDNA assemblies described above. These sites were then used to clone the cDNA assembly into the binary expression vector pAG01 (Gomord *et al*., 2020). pAG01 vector also contained an expression cassette for the silencing inhibitor p19. The vectors were then used to transform *Agrobacterium tumefaciens* strain LBA4404.

#### Plant cultivation and transient expression

Seeds of wild type *N. benthamiana* or of the FX‐KO Line of *N. benthamiana* deficient in plant‐specific α‐1,3‐fucosyltransferase and β‐1,2‐xylosyltransferase activities (Jansing *et al*., 2019) were sown in coco fibre plugs. As illustrated in Figure 1a, the seedlings were grown for 14 days in a hydroponic system under continuous LED lighting and then transferred to larger hydroponic tanks containing a nutrient medium under LED lighting at 26 °C and a 16 h/8 h day–night regimen where they were allowed to develop for 14 additional days. At the end of this period, their aerial part was immersed in a suspension of agrobacteria carrying the binary vector (the inoculum). The inoculum was then infiltrated in leaves by two cycles of vacuum (−0.8 Bar)/release. Following infiltration, plants were transferred to new hydroponic tanks.

*N. benthamiana* leaves were harvested 3–4 days post‐infiltration and extracted using a juicer (Angel 5500). After filtration on a combination of nylon filters, the extracts were used for magnetic purification of plant‐made mAbs.

#### Antibody extraction and magnetic purification

In the present study, 0.8–0.9 L of crude extract was typically obtained from 1 kg of *N. benthamiana* leaves. After filtration combination of nylon filters having a pore size from 40 μm to 25 μm and pH adjustment at pH 7.5 with Tris 2 M, this extract was incubated for 1 h in 1 L polystyrene Corning bottles with 8 mL (sedimented bead) of Protein A Mag Sepharose Xtra (Cytiva). Then, the incubated solution was transferred in a beaker and the magnetic bead was sedimented in a few seconds at the bottom by a magnetic flux of 0.3 T induced by a Neodymium magnet as illustrated in Figure 1a. The supernatant was discarded, and the magnetic bead was resuspended four times in 100 mL of 50 mM Tris–HCl, 125 mM NaCl buffer, pH7.5 (TBS) and then three times in 100 mL of TBS/10. Finally, Protein A magnetic beads were resuspended four times in 5 mL of 0.1 M Glycine buffer, pH 2.3 for mAb elution. The eluate was immediately neutralized by addition of Tris 2 M. Thus, starting from 1 kg of tobacco leaves the purified antibody was obtained in 20 mL of eluate, only 90 min after biomass collection (Faye *et al*., 2024).

#### SDS‐PAGE analysis

Protein samples were heated at 90 °C for 10 min in denaturation buffer A (Tris 62.5 mM, pH 6.8, containing 10% glycerol, 1% SDS and 2% β‐mercaptoethanol). Then protein samples were centrifuged at 10000xg for 5 min before loading on gels. SDS‐PAGE was performed on 16% or 4%–20% polyacrylamide Tris‐Glycine gels (Novex WedgeWell, XP0016BOX). For control of M15 and S15 integrity and purity, electrophoretic separation was performed under reducing or non‐reducing conditions and gels were silver‐stained.

#### N‐linked glycan analysis

Mass spectrometry was used to analyse the *N*‐linked glycosylation profiles of M15 and S15 and their HDEL, YTE and FX variants.

These mAbs were digested in‐solution, S‐alkylated with iodoacetamide and digested with Trypsin (Promega). Additionally, the samples were de‐glycosylated using PNGase A to estimate the proportion of not glycosylated peptide and analysed by LC–MS. The digested samples were loaded on a nanoEase C18 column (nanoEase M/Z HSS T3 Column, 100 Å, 1.8 μm, 300 μm × 150 mm, Waters) using 0.1% formic acid as the aqueous solvent. A gradient from 1% B (B: 80% Acetonitrile, 0.1% FA) to 40% B in 50 min was applied, followed by a 10 min gradient from 40% B to 95% B that facilitates elution of large peptides, at a flow rate of 6 μL/min. Detection was performed with an Orbitap MS (Exploris 480, Thermo) equipped with the standard H‐ESI source in positive ion, DDA mode (=switching to MSMS mode for eluting peaks). MS‐scans were recorded (range: 350–1200 Da) and the 20 highest peaks were selected for fragmentation. Instrument calibration was performed using Pierce FlexMix Calibration Solution (Thermo Scientific). The possible glycopeptides were identified as sets of peaks consisting of the peptide moiety and the attached N‐glycan varying in the number of HexNAc units, hexose, deoxyhexose and pentose residues. The theoretical masses of these glycopeptides were determined with a spread sheet using the monoisotopic masses for amino acids and monosaccharides. Manual glycopeptide searches were made using Freestyle 1.8 (Thermo). For the quantification of the different glycoforms, the peak areas of EICs (Extracted Ion Chromatograms) of the first four isotopic peaks were summed, using the quantification software Skyline (University of Washington).

#### COVID‐19 model in Syrian hamsters

Female golden Syrian hamsters, aged 6–7 weeks, were housed in the ABSL‐4 facility of the Galveston National Laboratory. The animal protocol # 2004049 was approved by the Institutional Animal Care and Use Committee (IACUC) of the University of Texas Medical Branch at Galveston (UTMB).

Golden Syrian hamsters were randomly assigned to two groups of *n* = 5 and microchipped 24 h before SARS‐CoV‐2 challenge. On the day of challenge, hamsters were anaesthetised with ketamine/xylazine and challenged by the intranasal route with 10 000 PFU of SARS‐CoV‐2 diluted in sterile PBS in the total volume 100 μL. Body weight and body temperature were measured each day, starting at day 0. Next day hamsters were treated with 10 mg kg‐1 of COVA2‐15 diluted in 0.5 mL of sterile PBS or PBS only in control group via intraperitoneal route. All animals were euthanized 72 h post‐infection (day 3), and terminal blood collected. Hamsters were euthanized with an overdose of anaesthetic (isoflurane or ketamine/xylazine) followed by bilateral thoracotomy. Lungs were harvested for all groups. Right lungs were frozen in 5 mL L15 media. Tissue sections were homogenized in bead beater tubes, weighed, and supernatants were titrated per standard protocol. Briefly, 10‐fold dilutions of supernatants at 100 μL per well were placed atop of Vero‐E6 monolayers in 96‐well plates and, after 1 h of incubation, supernatants were replaced by methyl cellulose overlay, incubated for 3 days at 5% CO_2_ and 37 °C. The plates were fixed with formalin, removed from BSL‐4 according to the approved protocol, and plaques counted to determine the titres.

Sera collected from animals were tested for neutralizing capabilities against SARS‐CoV‐2. Neutralization assay medium is MEM (Gibco) supplemented with 2% FBS (Gibco), 50 μg/mL gentamicin sulphate (Corning) and 5 mM Hepes buffer (Corning). Aliquots of mNeonGreen reporter SARS‐CoV‐2 were pre‐incubated for 1 h in 5% CO2 at 37 °C with initial dilution 1:20 following by 2× fold dilutions and inoculated into Vero‐E6 monolayers in black polystyrene 96‐well plates with clear bottoms (Corning). The final amount of virus was 200 PFU/well, which was the starting concentration of serum at 1:20 dilution. After 2 days of incubation, the fluorescence intensity of infected cells was measured at a 488 nm wavelength using a Cytation 5 Cell Imaging Multi‐Mode Reader (Biotek). The signal readout was normalized to virus control aliquots with no mAb added and was presented as the percentage of neutralization. The concentration of serum required to reduce half the maximal infectivity (EC_50_) was calculated with Prism version 9, GraphPad Software. The detection limit is serum dilution 1:10.

#### Spike‐binding ELISA

The 96‐well high binding ELISA microplates (Greiner) were loaded with 2 μg/mL SARS‐CoV‐2 Spike Wuhan in PBS overnight at 4 °C. Next, plates were blocked for 1 h with PBS/1% BSA at RT. Antibody dilutions were prepared in PBS/0.5% BSA starting 5 nM serially diluted 10‐fold, added onto plates, and incubated for 1 h at RT. Secondary antibody, goat anti‐human IgG (H + L) peroxidase‐labelled (SeraCare), were prepared by diluting 1:3000 with PBS/0.5% BSA and added onto plates for 1 h at RT in the dark. After incubation, developing solution (0.1 M NaAc +0.1 M citric acid +1% TMB + 0.01% H_2_O_2_) was added, incubated for 5 min in the dark and stopped with 0.8 M H_2_SO_4_ and signal was measured after 2 min. Optical density, OD_450_, was measured using SPECTROstar Nano Microplate Reader (BMG LabTech). Each subsequent steps, plates were washed 5x with TBS‐T (1X Tris‐buffered saline supplemented with 0.05% Tween‐20) with AquaMax^®^ 4000 Microplate Washer (Molecular Devices).

#### Spike‐binding biolayer interferometry

BLI experiments were performed as previously described (Caniels *et al*., 2021) on an Octet K2 (ForteBio). Briefly, Ni‐NTA biosensors (ForteBio) were loaded with 5 μg/mL of hexahistidine‐tagged SARS‐CoV‐2 Spike or SARS‐CoV‐2 RBD in running buffer (PBS, 0.02% Tween 20, and 0.1% bovine serum albumin) for 300 s. After the biosensors were washed in a well containing running buffer to remove the excess protein for 60 s, the biosensors were dipped in a well containing mAbs at a concentration of 100 nM for 120 s to measure association. Subsequently, dissociation was measured by dipping the biosensors into a well containing running buffer.

#### Neutralization

Neutralization activity was tested using a pseudovirus neutralization assay, as previously described (Caniels *et al*., 2021). Shortly, pseudoviruses were produced by co‐transfecting the pCR3 SARS‐CoV‐2‐SΔ19 (Wuhan‐Hu‐1, GenBank MN908947.3 with amino acid substitution D614G) expression plasmid with the pHIV‐1NL43 ΔEnv‐NanoLuc reporter virus plasmid in HEK293T cells (ATCC, CRL‐11268), as previously described (Schmidt *et al*., 2020). Supernatant was harvested 48 h post transfection and stored at −80 °C until further use. Then, HEK293T/ACE2 cells were seeded at a density of 20 000 cells/well in a 96‐well plate coated with 50 μg/mL poly‐L‐lysine one day prior to the start of the neutralization assay. Antibodies were serially diluted in cell culture medium (DMEM (Gibco), supplemented with 10% FBS, penicillin (100 U/mL), streptomycin (100 μg/mL) and GlutaMax (Gibco)), subsequently mixed in a 1:1 ratio with pseudovirus and incubated for 1 h at 37 °C. Subsequently, these mixtures were added to the cells in a 1:1 ratio and incubated for 48 h at 37 °C, followed by cell lysis to measure the luciferase activity in cell lysates using the Nano‐Glo Luciferase Assay System (Promega) and GloMax system (Turner BioSystems). Relative luminescence units (RLU) were normalized to the positive control wells where cells were infected with pseudovirus in the absence of antibodies. The neutralization titers (IC_50_) were determined as the antibody concentration at which infectivity was inhibited by 50%, respectively, using a non‐linear regression curve fit (GraphPad Prism software version 8.3).

#### Fc gamma receptor ELISA

The 96‐well high binding standard ELISA microplates were loaded with 2 μg/mL SARS‐CoV‐2 Spike Wuhan in PBS overnight at 4 °C. Next, plates were and blocked for 1 h with PBS/1% BSA at RT. Antibody dilutions were prepared in PBS/0.5% BSA starting 150 nM serially diluted three‐fold, added onto plates, and incubated for 1 h at RT. Biotinylated FcγRIIA and FcγRIIIA were prepared in PBS/0.5% BSA at 0.5 μg/mL and added onto plates, incubated for 1 h at RT. Next, high sensitivity streptavidin‐HRP was diluted 1:2000 in PBS/0.5% BSA (BioLegend) were added onto the plates and incubated for 1 h at RT in the dark. After incubation, developing solution (0.1 M NaAc +0.1 M citric acid +1% TMB + 0.01% H_2_O_2_) was added, incubated for 5 min in the dark and stopped with 0.8 M H_2_SO_4_ and signal was measured after 2 min. Optical density, OD_450_, was measured using SPECTROstar Nano Microplate Reader (BMG LabTech). For each subsequent step, plates were washed 5x with TBS‐T (1X Tris‐buffered saline supplemented with 0.05% Tween‐20) with AquaMax^®^ 4000 Microplate Washer (Molecular Devices).

#### NK cell activation

NK cell activation assay was essentially performed as previously described (van der Straten 2024). Briefly, half‐area microplates (Greiner) were coated overnight at 4 °C with 2 μg/mL SARS‐CoV‐2 Spike Wuhan in PBS. Plates were blocked with PBS/1% BSA and subsequently washed three times with TBS. Then, antibodies were diluted to 50 nM in PBS/1% BSA and incubated in the plates for 1 h at 37 °C. Plates were washed again three times with TBS before adding 5 × 10^4^ NK cells per well in the presence of 10 ng/mL IL‐15 and 5 μg/mL Brefeldin A. NK cells were isolated from healthy donor PBMCs one day prior to NK cell activation assay and were cultured overnight in the presence of 10 ng/mL IL‐15 to support survival and viability. Plates were incubated for 3 h at 37 °C before NK cells were transferred to a 96‐well V‐bottom microplate and stained with anti‐CD16 BV421 (Clone 3G8; 1:500; BioLegend), anti‐CD107 APC (Clone H4A3; 1:1000; Biolegend) and fixable viability dye ef780 (Invitrogen; 1:2000). After 1 h incubation at 4 °C, cells were washed 3x with FACS buffer (PBS + 2% FCS), and subsequently fixed with the Fixation/Permeablization Kit (BD biosciences). After fixation and permeabilization, NK cells were stained intracellularly with anti‐IFNγ PE (Clone B27; 1:500; BioLegend) antibody in Wash/Perm buffer (BD biosciences). After intracellular stain, cells were washed twice in wash buffer and then resuspended in 150 μL FACS buffer for flow cytometry analysis. Samples were measured using a BD FACSymphony A1 Cell Analyser and analysed with FlowJo.

#### ADCP

This assay was performed following previously described methods (Claireaux *et al*., 2022). In summary, fluorescent NeutrAvidin beads (Invitrogen) were incubated overnight at 4 °C in PBS with biotinylated SARS‐CoV‐2 Spike Wuhan protein (10 μg/5 μL beads suspension). Subsequently, the beads underwent two washes using PBS 2% bovine serum albumin (BSA). The coated beads were resuspended in PBS 2% BSA at a dilution of 1:500. 50 μL of the bead suspension was placed in a V‐bottom 96‐well plate and serial dilutions of the antibodies were added, followed by a 2 h incubation at 37 °C. Next, the plates were washed and 5 × 10^4^ THP‐1 effector cells (ATCC) were added to each well. To promote bead to cell contact, plates were quickly spun down before a 5 h incubation at 37 °C. After incubation, the cells were washed and stained with a fixable viability dye ef780 (BioLegend) at 4 °C for 30 min. After incubation, the cells were washed twice, resuspended in PBS 2% FCS, and analysed by flow cytometry to determine phagocytic activity, represented as the mean fluorescent intensity (beads positive cells × mean MFI FITC).

#### FcRn binding

The Lumit® FcRn Binding Immunoassay was performed following manufacturer's instructions (Promega). Briefly, the assay involves three steps. In the first step, 25 μL of IgG‐LgBiT solution was mixed with 25 μL of the Fc fusion proteins in a white, 96‐well, non‐binding plate (Corning). In the second step, 50 μL of cFcRn‐SmBiT was added to each well containing IgG and IgG‐LgBiT and the plate incubated for 30 min at RT. In the final step, 25 μL of Lumit detection solution and signal was allowed to stabilize for 3–5 min, and the bioluminescence signal (relative light units [RLU]) was measured in a Berthold LB940. All the dilutions were made in PBS containing 10% of adjusting buffer (0.5 M citrate buffer containing 50% super‐block) to decrease the final pH to a value of 6. All the experiments were run in duplicate or triplicate. Normalized RLU data were generated by assigning 100% to the maximum bioluminescent signal obtained in absence of the IgG and then calculating percentage drop in signal in the presence of an IgG. Inhibition curves were generated by plotting normalized RLU as a function of log value of the IgG concentration (in μg/mL) and fitted to a four‐parametric equation with 1/y^2^ weightage using Origin Pro 8.5 (OriginLab). IC_50_ values that are equivalent to apparent affinity values (KD) were used when comparing data generated using biosensor and reported in the literature.

## Conflict of interest

Véronique Gomord is co‐founder, board member, and CSO of Angany Inc and CEO of Angany InnovationL, Louis‐Philippe Vezina is co‐founder of Angany Inc, Réjean Desgagnés, Loïc Faye, Anne‐Catherine Fitchette, Virginie Catala‐Stordeur, Bertrand Morel, Lucie Mirande, Guillaume Beauverger are employees of Angany Inc. or Angany Innovation.

## Author contributions

Conceptualization and design of study: MJvG, RWS, GdB, ABW, IAW, VG, L‐PV. Experimental investigation: SWdT, AIS, JCU, MY, XZ, HLT, JAB, MP, TC, RD, LF, A‐CF, VS, BM, LM, CG‐G, NAK, AB and GB. Data analysis: SWdT, TB, VG, LF, BM, CG‐G, GO, NAK, AB. Writing original draft: SWdT, LF, TB. Editing and revisions: SWdT, LF, MJvG, RWS, AIS, JCU, MY, IAW, VG, GO, NAK.

## Supporting information


**Table S1** X‐ray data collection and refinement statistics.
**Table S2** Cryo‐EM data collection, processing and model refinement statistics.
**Table S3** *N*‐linked glycosylation forms ESI‐MS.

**Figure S1** Therapeutic activity of plant‐produced SARS‐CoV‐2 antibody COVA2‐15. (a) Side view (left panel) and top view (middle panel) of 3.9 Å cryo‐EM structure of three COVA2‐15 Fab domains in complex with SARS‐CoV‐2 Wuhan spike. Right panel illustrates an overlay of the 3.9 Å cryo‐EM structure (with only a single Fv:spike protomer displayed for clarity) and the 3.4 Å crystal structure, validating the structural model and COVA2‐15 interaction. (b) Body weight kinetics is plotted for each group of hamsters receiving COVA2‐15, M15, S15 or no Ab treatment. (c) Neutralization activity in serum against SARS‐CoV‐2 two days after administration of therapeutic antibody. No significant differences were observed between groups in terms of body weight or neutralizing activity.
**Figure S2** Fc gamma receptor binding of engineered plant‐produced COVA2‐15 variants. (a) SDS‐Page gel electrophoresis showing plant‐produced S15 and M15 engineered variants under reducing and non‐reducing conditions. Similar purity and homogeneity is observed for engineered variants compared to S15 and M15 WT productions. (b) Spike binding and neutralization by Fc and glyco‐engineered plant‐produced biosimilar antibodies. A representative experiment is shown for spike binding in ELISA and neutralization activity in the 293 T‐ACE2 neutralization assay. (c) Binding of plant‐produced S15 and M15 variants to FcγRIIa‐dimer and FcγRIIIa‐dimer, as determined by an FcγR‐dimer ELISA. This is a representative experiment and the AUC values of the binding curves are plotted in Figure 2d.
**Figure S3** (a) Fc effector function of engineered plant‐produced COVA2‐15 variants. Bar graph showing mean + SEM (*n* = 3) CD16 shedding by primary NK cells in response to plant‐produced S15 and M15 variants. (b) Spearman's correlation between CD16 shedding and NK‐cell activation for two donors (left and middle graph) and between FcγRIIIa binding and NK cell activation. (c) Phagocytic activity by THP‐1 cells in response to a serial dilution of human and S15 (left panel) and M15 (right panel) plant‐produced COVA2‐15 variants.

## Data Availability

The data that supports the findings of this study are available in the supplementary material of this article.
