# Supplementary material for: Plant‐produced SARS‐CoV‐2 antibody engineered towards enhanced potency and in vivo efficacy
Source: Plant Biotechnol J. 2024 Nov 19;23(1):4–16. doi: 10.1111/pbi.14458 (PMC11672753; doi:10.1111/pbi.14458)
Supplement: Supplementary file 1 — Table S1 X‐ray data collection and refinement statistics. Table S2 Cryo‐EM data collection, processing and model refinement statistics. Table S3 N‐linked glycosylation forms ESI‐MS. [file PBI-23-4-s002.docx]

**Supplementary Information**

**Plant-produced SARS-CoV-2 antibody engineered towards enhanced potency and in vivo efficacy**

**Table S1. X-ray data collection and refinement statistics**

| **Data collection** | COVA2-15 + SARS-CoV-2 RBD | |
| --- | --- | --- |
| Beamline | APS23ID-D | |
| Wavelength (Å) | 0.97934 | |
| Space group | P 1 2_1_ 1 | |
| Unit cell parameters |  | |
| a, b, c (Å) | 101.1, 218.7, 122.7 | |
| α, β, γ (°) | 90, 112.3, 90 | |
| Resolution (Å) ^a^ | 50.0-3.40 (3.46-3.40) | |
| Unique reflections ^a^ | 68,687 (3,372) | |
| Redundancy ^a^ | 6.8 (6.8) | |
| Completeness (%) ^a^ | 100 (100) | |
| <I/σ_I_> ^a^ | 7.5 (1.1) | |
| *R*_sym_^b^ (%) ^a^ | 20.8 (>100) | |
| *R*_pim_^b^ (%) ^a^ | 8.7 (55.4) | |
| CC_1/2_^c^ (%) ^a^ | 98.4 (67.7) | |
| **Refinement statistics** | |  |
| Resolution (Å) | 33.1-3.70 | |
| Reflections (work) | 67,804 | |
| Reflections (test) | 3,273 | |
| *R*_cryst_^d^ / *R*_free_^e^ (%) | 24.2/29.5 | |
| No. of copies in ASU | 6 | |
| No. of atoms | 29,362 | |
| Fab | 20,148 | |
| RBD | 9,214 | |
| Average *B-*values (Å^2^) | 114 | |
| Fab | 109 | |
| RBD | 126 | |
| Wilson *B*-value (Å^2^) | 94 | |
| **RMSD from ideal geometry** | |  |
| Bond length (Å) | 0.002 | |
| Bond angle (^o^) | 0.50 | |
| **Ramachandran statistics (%) ^f^** | |  |
| Favored | 97.7 | |
| Outliers | 0.0 | |
| **PDB code** | 9B82 | |

^a^ Numbers in parentheses refer to the highest resolution shell.

^b^ *R*_sym_ = Σ*_hkl_* Σ*_i_* | I*_hkl,i_* - <I*_hkl_*> | / Σ*_hkl_* Σ*_i_* I*_hkl,i_* and R*_pim_* = Σ*_hkl_* (1/(n-1))^1/2^ Σ*_i_* | I*_hkl,i_* - <I*_hkl_*> | / Σ*_hkl_* Σ*_i_* I*_hkl,i_*, where I*_hkl,i_* is the scaled intensity of the i^th^ measurement of reflection h, k, l, <I*_hkl_*> is the average intensity for that reflection, and *n* is the redundancy.

^c^ CC_1/2_ = Pearson correlation coefficient between two random half datasets.

^d^ *R*_cryst_ = Σ*_hkl_* | *F*_o_ - *F*_c_ | / Σ*_hkl_* | *F*_o_ | x 100, where *F*_o_ and *F*_c_ are the observed and calculated structure factors, respectively.

^e^ *R*_free_ was calculated as for *R*_cryst_, but on a test set comprising 5% of the data excluded from refinement.

^f^ From MolProbity (72)

**Table S2. Cryo-EM data collection, processing and model refinement statistics**

| **Map** | **SARS-CoV-2-6Pmut7 Spike + COVA2-15 Fab** |
| --- | --- |
| EMDB | EMD-43794 |
| **Data collection** |  |
| Microscope | TFS Arctica |
| Voltage (kV) | 200 |
| Detector | Gatan K2 Summit |
| Recording mode | Counting |
| Nominal magnification | 36,000 |
| Movie micrograph pixelsize (Å) | 1.15 |
| Dose rate (e^-^/[(camera pixel)*s]) | 5.87 |
| Number of frames per movie micrograph | 45 |
| Frame exposure time (ms) | 250 |
| Movie micrograph exposure time (s) | 11.25 |
| Total dose (e^-^/Å^2^) | 50 |
| Defocus range (µm) | -0.7 to -2.0 |
| **EM data processing** |  |
| Number of movie micrographs | 2,770 |
| Number of molecular projection images in map | 25,728 |
| Symmetry | C3 |
| Map resolution (FSC 0.143; Å) | 3.9 |
| Map sharpening B-factor (Å^2^) | -100 |
| **Structure Building and Validation** |  |
| *Number of atoms in deposited model* |  |
| SARS-CoV-2 S | 25,557 |
| Fab Fv | 5,487 |
| glycans | 882 |
| MolProbity score | 0.94 |
| Clashscore | 0.81 |
| Map correlation coefficient | 0.75 |
| d FSC model (0.5) | 4.2 |
| EMRinger score | 1.24 |
| *RMSD from ideal* |  |
| Bond length (Å) | 0.006 |
| Bond angles (˚) | 1.043 |
| *Ramachandran plot* |  |
| Favored (%) | 96.88 |
| Allowed (%) | 3.12 |
| Outliers (%) | 0.00 |
| Side chain rotamer outliers (%) | 0.09 |
| Cβ outliers (%) | 0.00 |
| PDB | 9aru |
|  |  |
|  |  |

**Table S3. *N*-linked glycosylation forms ESI-MS**

|  |  | **M15 (plant)** | | | | **S15 (plant)** | | | | **Human** |
| --- | --- | --- | --- | --- | --- | --- | --- | --- | --- | --- |
| **Glycoform** |  | **WT** | **YTE** | **HDEL** | **FX** | **WT** | **YTE** | **HDEL** | **FX** | **COVA2-15** |
| Not Glyc. |  | 7,75 | 27,25 | 9,1 | 16,04 | 5,99 | 15,19 | 6,29 | 14,96 | 0,16 |
| GnGn |  | 5,23 | 7,19 | 0,17 | 52,49 | 6,31 | 10,23 | 0,18 | 53,94 | 0,29 |
| GnGnF/X/FX |  | 73,09 | 50,34 | 1,78 | 0 | 72,17 | 54,22 | 0,9 | 0 | 62,12 |
| GnM/F/X/UX |  | 2,54 | 10,15 | 0,25 | 0 | 3,92 | 12,41 | 0,19 | 0 | 4,69 |
| GnM |  | 0 | 0 | 0 | 7,55 | 0 | 0 | 0 | 6,58 | 0 |
| MMXF |  | 2,45 | 1,22 | 0,06 | 0 | 5,17 | 1,48 | 0,13 | 0 | 0 |
| MM |  | 0 | 0 | 0 | 1,08 | 0 | 0 | 0 | 0,58 | 0 |
| Man |  | 8,94 | 3,85 | 88,64 | 22,84 | 6,44 | 6,47 | 92,31 | 23,94 | 2,28 |
| AA/AAF/AGn/AGnF |  | 0 | 0 | 0 |  | 0 | 0 | 0 | 0 | 29,87 |
| NaA/NaAF/NaNa/ |  | 0 | 0 | 0 |  | 0 | 0 | 0 | 0 | 0,59 |

*N-glycans are defined by it’s terminal residues (71); Gn= terminal GlcNac, M= terminal mannose, A= terminal galactose, Na= terminal Sialic acid, F= terminal fucose, X= terminal Xylose, Man= High mannose glycan (Man7-Man9)*

**SI Figure 1. Therapeutic activity of plant-produced SARS-CoV-2 antibody COVA2-15. (A)** Side view (left panel) and top view (middle panel) of 3.9 Å cryo-EM structure of three COVA2-15 Fab domains in complex with SARS-CoV-2 Wuhan spike. Right panel illustrates an overlay of the 3.9 Å cryo-EM structure (with only a single Fv:spike protomer displayed for clarity) and the 3.4 Å crystal structure, validating the structural model and COVA2-15 interaction. **(B)** Body weight kinetics is plotted for each group of hamsters receiving COVA2-15, M15, S15 or no Ab treatment. **(C)** Neutralization activity in serum against SARS-CoV-2 two days after administration of therapeutic antibody. No significant differences were observed between groups in terms of body weight or neutralizing activity.

**SI Figure 2. Fc gamma receptor binding of engineered plant-produced COVA2-15 variants. (A)** SDS-Page gel electrophoresis showing plant-produced S15 and M15 engineered variants under reducing and non-reducing conditions. Similar purity and homogeneity is observed for engineered variants compared to S15 and M15 WT productions. (**B**) Spike binding and neutralization by Fc and glyco-engineered plant-produced biosimilar antibodies. A representative experiment is shown for spike binding in ELISA and neutralization activity in the 293T-ACE2 neutralization assay. (**C**) Binding of plant-produced S15 and M15 variants to FcγRIIa-dimer and FcγRIIIa-dimer, as determined by an FcγR-dimer ELISA. This is a representative experiment and the AUC values of the binding curves are plotted in Figure 2D.

**SI Figure 3. (A) Fc effector function of engineered plant-produced COVA2-15 variants.** Bar graph showing mean + SEM (n=3) CD16 shedding by primary NK cells in response to plant-produced S15 and M15 variants. (**B**) Spearman’s correlation between CD16 shedding and NK-cell activation for two donors (left and middle graph) and between FcγRIIIa binding and NK cell activation. (**C**) Phagocytic activity by THP-1 cells in response to a serial dilution of human and S15 (left panel) and M15 (right panel) plant-produced COVA2-15 variants.
