# Supplementary material for: Plant‐produced SARS‐CoV‐2 antibody engineered towards enhanced potency and in vivo efficacy
Source: Plant Biotechnol J. 2024 Nov 19;23(1):4–16. doi: 10.1111/pbi.14458 (PMC11672753; doi:10.1111/pbi.14458)

SI Figure 1:

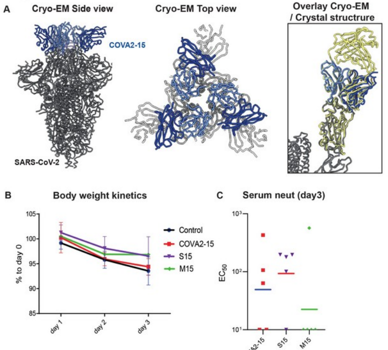

SI Figure 2:

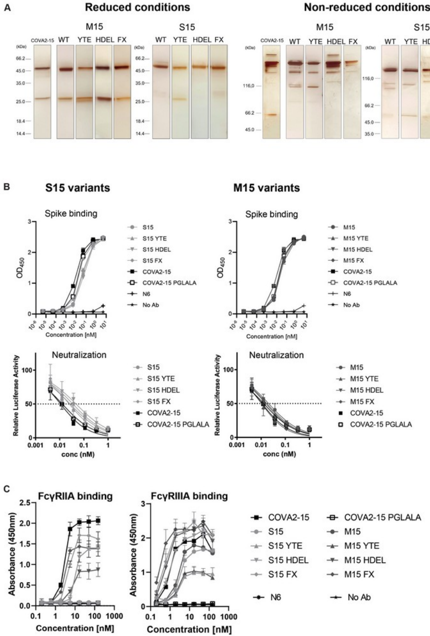

SI Figure 3:

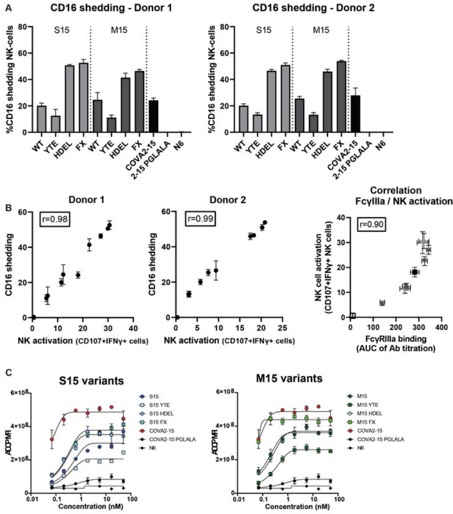

Supplement: Supplementary file 2 [file PBI-23-4-s001.docx]
